# Characterization of the atypical antipsychotic drug aripiprazole cytotoxicity in the neutrophil model cell line HL-60

**DOI:** 10.1371/journal.pone.0318878

**Published:** 2025-02-12

**Authors:** Courtney A. Swain, Emily J. Robbs, Lindsay Verma, Heaven Brandt, Alexandra L. Seppaenen, Peter J. Cavnar

**Affiliations:** 1 Department of Pathology, The University of Alabama at Birmingham, Birmingham, Alabama, United States of America; 2 Department of Biology, University of West Florida, Pensacola, Florida, United States of America; NYU Grossman School of Medicine: New York University School of Medicine, UNITED STATES OF AMERICA

## Abstract

Atypical antipsychotics are widely used for the treatment of mental and behavioral disorders such as bipolar disorder, obsessive-compulsive disorder, and schizophrenia. However, these drugs can occasionally induce neutropenia or agranulocytosis, characterized by a significant reduction in circulating neutrophils, the primary white blood cells responsible for immune responses. This drug-induced neutropenia poses a considerable risk of life-threatening infections. However, the precise mechanism by which atypical antipsychotics induce neutropenia remains unclear. This study investigates the effects of four atypical antipsychotics, namely – aripiprazole, clozapine, olanzapine, and quetiapine – on the human neutrophil model cell line HL-60. These drugs, which modulate dopamine receptor signaling alongside other mechanisms, were analyzed for their effects. Among these, aripiprazole – but not the others – uniquely induced apoptosis in a dose-dependent manner, accompanied by an increased expression of pro-apoptotic genes – BAK, BCL10, and caspase-3. Moreover, our study elucidates that while differentiated HL-60 cells express D1-like and D2-like dopamine receptors, aripiprazole’s cytotoxic effects appear to operate through dopamine-independent pathways and significantly reduce phosphorylated Src family kinase levels. Our results align with previous studies suggesting that aripiprazole exhibits cytotoxic properties in neutrophils. Nevertheless, further investigations are warranted to investigate the mechanisms underlying aripiprazole-induced apoptosis in neutrophils.

## Introduction

Neutrophils, the most abundant white blood cells comprising 60–70% of the total leukocyte population, are critical components of innate immunity as the first responders to inflammation caused by acute injury or infection [1]. Defects or loss-of-function mutations in neutrophil-specific processes can negatively affect neutrophil homeostasis which can lead to conditions such as neutropenia, a significant reduction of neutrophils in peripheral blood [2]. Neutropenia can increase one’s susceptibility to life-threatening infections and can be induced by drug interactions [3]. Chemotherapy drugs are a common cause of drug-induced neutropenia, in addition to drugs that treat hyperthyroidism, blood thinners, and atypical antipsychotic medications to treat mental and behavioral disorders such as schizophrenia and bipolar disorder [4]. The exact mechanisms for many cases of drug-induced neutropenia are not well known.

In this study, we characterized the effects of the atypical antipsychotic drugs, namely – aripiprazole, clozapine, olanzapine, and quetiapine – which act on dopamine receptor signaling in addition to other mechanisms of action, on the human neutrophil model cell line HL-60 cells, a promyelocytic leukemia cell line that can be terminally differentiated into “neutrophil-like” cells by all-trans retinoic acid (ATRA) or dimethyl sulfoxide (DMSO) treatment [5]. Dopamine is an important neurotransmitter in the central nervous system (CNS) and peripheral tissues that regulate functions like motivation and reward, cognition, and movement [6]. There are five dopamine receptors categorized into D1-like receptors (dopamine receptors D1R and D5R) based on their ability to activate Gαs and cyclic-AMP (cAMP) signaling, and D2-like receptors (dopamine receptors D2R, D3R, and D4R), which activate Gαi and inhibit adenylyl cyclase and cAMP synthesis [7]. Surprisingly, neutrophils express dopamine receptors. For example, high surface expression of dopamine D3R and D5R, low expression of D2R and D4R, and no expression of D1R were reported from isolated human peripheral blood [8]. Futhermore, molecular analysis detected the presence of D1R, D2R, D3R, and D5R in ATRA-differentiated HL-60 cells [9]. Finally, intracellular dopamine in neutrophils has been reported, and evidence suggests that neutrophils can both synthesize and metabolize dopamine and its metabolites [10].

Atypical antipsychotics are a class of drugs used to treat psychiatric disorders such as schizophrenia and bipolar disorder. Clozapine, like most atypical antipsychotics, is a dopamine receptor antagonist, binding to D4R with a higher affinity than D2R [11]. However, several reports demonstrate the risk of developing agranulocytosis in patients taking clozapine [12–14]. It remains unclear exactly how clozapine causes neutropenia. Several studies suggest clozapine exhibits cytotoxic effects on neutrophils due to clozapine being metabolized to a nitrenium cation, which induces oxidative stress and apoptosis, independent of dopamine receptor mechanisms [15–18]. Olanzapine and quetiapine similarly exert their effects through dopamine receptor antagonism. Several case studies document olanzapine’s ability to induce neutropenia [19–24]. A literature review reports numerous published case studies describing quetiapine-induced neutropenia or agranulocytosis in patients [25]. In-vitro, clozapine, olanzapine, and quetiapine have exhibited dose-dependent toxicity in granulocyte precursors, which suggests these atypical antipsychotic drugs may induce neutropenia through effects on neutrophil differentiation [26].

Aripiprazole is a commonly prescribed antipsychotic for schizophrenia, bipolar disorder, obsessive-compulsive disorder, and irritability associated with autism, which makes it one of the leading atypical antipsychotics used in adolescents [27]. Compared to the previously described atypical antipsychotics, aripiprazole is unique because it is a partial agonist of D2R, D3R, and D4R receptors, as well as a partial activator of serotonin receptors [11]. Like the other atypical antipsychotics described, case studies report aripiprazole-induced neutropenia and leukocytopenia [28–30]. Furthermore, aripiprazole affects the mitochondria by inhibiting complex I- and II-linked respiration [31]. Interestingly, aripiprazole induced cytotoxicity in tumor cell lines, which exhibited increased expression of pro-apoptotic genes and significantly reduced Src kinase activity [32,33]. Due to limited data regarding aripiprazole-induced neutropenia, aripiprazole is recommended as a therapeutic alternative in cases of neutropenia or leukopenia induced by other antipsychotic agents [34]. However, there is a significant lack of studies that characterize the effects of aripiprazole on neutrophils.

## Materials and Methods

### Atypical antipsychotics and dopamine agonists and antagonists

Clozapine (Clozaril®; 3-chloro-6-(4-methylpiperazin-1-yl)-11H-benzo[b][1,4]benzodiazepine), olanzapine (Zyprexa®; 2-methyl-4-(4-methylpiperazin-1-yl)-10H-thieno[2,3-b][1,5]benzodiazepine), aripiprazole (Abilify®; 7-[4-[4-(2,3-dichlorophenyl)piperazin-1-yl]butoxy]-3,4-dihydro-1H-quinolin-2-one), quetiapine hemifumarate (Seroquel®; 2-[2-(4-dibenzo[b,f][1,4]thiazepin-11-yl-1-piperazinyl)ethoxy]-ethanol hemifumarate)), flupentixol (4-[3-[2-(Trifluoromethyl)-9H-thioxanthen-9-ylidene]propyl]-1-piperazineethanol), S-(-)-eticlopride hydrochloride (S-(−)-3-Chloro-5-ethyl-N-[(1-ethyl-2-pyrrolidinyl)methyl]-6-hydroxy-2-methoxybenzamide hydrochloride), SKF38393 hydrochloride, quinpirole hydrochloride, 7-hydroxy-2-(di-n-prpylamino)tetralin hydrobromide, PD 168077 Maleate, SCH 23390 hydrochloride, and haloperidol were purchased from Sigma-Aldrich. All reagents were solubilized in DMSO at a 5 mM stock solution and stored at -20°C under desiccation.

### Cell culture

HL-60 cells were obtained from the American Type Culture Collection (ATCC) and maintained at a concentration between 0.1 – 1.0 x 10^6^ cells/mL in RPMI-1640 containing L-glutamine (HyClone™) supplemented with 10% fetal bovine serum (FBS; Corning) and 0.2% Normocin (Invivogen), at 37°C and 5% CO_2_. HL-60 cells were differentiated (dHL-60) by incubating 0.2 x 10^6^ cells/mL in complete media with 1.3% dimethyl sulfoxide (DMSO). Experiments involving dHL-60 cells were performed on cells five to seven days post-differentiation. dHL-60 differentiation status was confirmed by flow cytometry analysis of CD11b expression.

### XTT cell viability assay

Cell viability was quantified using the CyQUANT™ XTT Cell Viability Assay (ThermoFisher Scientific). 1 x 10^5^ dHL-60 cells were cultured in triplicate in a 96-well plate with complete media containing 10% or 2% FBS. Cells were treated with 0.1%, 0.4%, and 1% DMSO, or 1 μM, 20 μM, and 50 μM aripiprazole, clozapine, olanzapine, and quetiapine. In additional experiments, cells were treated with 0.4% DMSO or 20 μM of dopamine agonists and antagonists as indicated in Table 1 [7,11,35–41] for 48 hours at 37°C and 5% CO_2_. Afterward, cells were incubated with the CyQUANT™ XTT reagent for four hours following the manufacturer’s protocol. The XTT reagent is a tetrazolium-based compound that becomes reduced by actively respiring cells to produce a water-soluble orange-colored formazan product, which was measured by quantifying the absorbance at 450 nm with a multi-mode plate reader (PerkinElmer, Inc.). The average absorbances from triplicate technical replicates and at least three independent biological replicates were normalized to DMSO control treatment.

**Table 1:** Dopamine receptor subtype agonists and antagonists used in this study.

Compound	Dopamine Receptor Subtype	Agonist/Antagonist	Reference
7-OH-DPAT	D3R	Agonist	[35]
Eticlopride	D2R	Antagonist	[36]
Flupentixol	D1-/D2-like	Antagonist	[37]
Haloperidol	D2R, D3R, D4R	Antagonist	[38]
Olanzapine	D2R	Antagonist	[11]
PD 168077	D4R	Agonist	[39]
Quinpirole	D2-like	Agonist	[7]
SCH 23390	D1R, D5R	Antagonist	[40]
SKF 38393	D1-like	Agonist	[41]

### Annexin V/PI apoptosis assay

Detection of cell apoptosis was completed using the BD Pharmingen™ FITC Annexin V apoptosis detection kit I (BD Biosciences). For this assay, 1 x 10^6^ dHL-60 cells were cultured in a 12-well plate in 1 mL complete media containing 10% FBS and 0.1%, 0.2%, and 0.4% DMSO, or 1 μM, 10 μM, and 20 μM aripiprazole for 24 hours at 37°C and 5% CO_2._ For oxidized clozapine studies, the following protocol was adapted from a previous study [42]. Briefly, 1 x 10^6^ cells/mL were cultured with 0.4% DMSO, 10 μM hydrogen peroxide (H_2_O_2_), or 20 μM clozapine and 10 μM H_2_O_2_ combined for 12 hours at 37°C and 5% CO_2._ After treatment, cells were washed once in PBS and stained with Annexin V and propidium iodide (PI) according to the manufacturer’s directions. Apoptosis was analyzed using the BD Accuri C6 flow cytometer. Gating was established using non-stained, Annexin V-only, and PI-only controls before each replicate analysis. 10,000 cells were recorded from each condition per replicate. The average percentage of Annexin V-positive only cells, which corresponds to early apoptosis, and Annexin V/PI positive cells, which corresponds to late apoptosis, were graphed from three independent replicates.

### RT-qPCR of pro-apoptotic genes

RT-qPCR was performed to measure the effect of aripiprazole on BAK1, CASP3, and BCL10 mRNA expression levels relative to ACTB and RPL13A reference genes following previously published protocols [43]. dHL-60 cells were treated five days post-differentiation with 0.1%, 0.2%, and 0.4% DMSO, or 1 μM, 10 μM, and 20 μM aripiprazole for 24 hours at 37°C and 5% CO_2._ The cells were washed once with PBS, and total RNA was purified using the RNeasy Mini Kit (Qiagen, Inc.). cDNA was synthesized using the iScript cDNA synthesis kit (Bio-Rad Laboratories) from 400 ng of total RNA per reaction. No-RT controls were included as negative controls throughout the RT-qPCR procedure. The primer pairs used for qPCR reactions are listed in Table 2. Primer validation was performed before qPCR analysis, including temperature gradient and standard curve analyses. DNA gel electrophoresis was performed to verify single-target amplification. qPCR amplification efficiencies for all targets were 90% or greater, and all qPCR reactions were performed in triplicate. Analysis of relative expression levels was quantified using the 2^-ΔΔCq^ method from three biological replicates.

**Table 2:** RT-qPCR primers used for mRNA expression analysis in dHL-60 cells treated with aripiprazole.

Gene	Forward Primer	Reverse Primer
β - Actin (ACTB)	5’ – CTTCGCGGGCGACGAT – 3’	5’ – CCACATAGGAATCCTTCTGACC – 3’
Ribosomal protein L13a (RPL13A)	5’ – TAAACAGGTACTGCTGGGCCG – 3’	5’ – CTCGGGAAGGGTTGGTGTTC – 3’
Caspase-3 (CASP3)	5’ – TTTTTCAGAGGGGATCGTTG – 3’	5’ – TTTCGCCAAGAATAATAACCTGAAT – 3’
BCL2 antagonist/killer 1 (BAK1)	5’ – GATCCCGGCAGGCTGATCCC – 3’	5’ – CCTGGGCTACCTGCTCCTCAGA – 3’
BCL10	5’ – TTTCCTCAGTGCATTTGTGC – 3’	5’ – GCCTATACGACAATGGAGTGGAA – 3’

### Flow cytometry analysis of dopamine receptor surface expression

We characterized dopamine surface receptor expression in dHL-60 cells by indirect immunofluorescence and flow cytometry. The following primary antibodies were used to detect specific dopamine receptor subtypes: PE mouse anti-human Dopamine D1 receptor (BioLegend), rabbit anti-dopamine D2R (extracellular) receptor (Alomone labs), rabbit anti-dopamine D3R receptor (MilliporeSigma), rabbit anti-dopamine D4R receptor (Invitrogen), and rabbit anti-dopamine D5R receptor (Invitrogen). All primary antibodies used were commercially purchased and raised against dopamine receptor extracellular immunogens. All staining procedures were completed on ice. 1 x 10^6^ dHL-60 cells were pre-incubated with Fc receptor binding inhibitor polyclonal antibody (Invitrogen) for 20 minutes. Cells were then incubated with dopamine receptor-specific primary antibodies or equivalent concentrations of species-specific isotype controls for 30 minutes. After incubation, cells were washed three times in flow cytometry buffer (5% FBS, 0.5 mM EDTA, and 1% sodium azide in PBS). The cells were incubated with FITC goat anti-rabbit IgG (ThermoFisher Scientific) for 30 minutes and washed with flow cytometry buffer. Surface expression was characterized using a BD Accuri C6 flow cytometer (BD Biosciences, Inc.). Fluorescence histograms were generated from 10,000 cells per replicate, displaying fluorescence intensities of unstained, secondary only, isotype control, and dopamine receptor-specific primary antibodies. A total of three independent biological replicates were performed.

### Western blot analysis

dHL-60 cells were treated with 0.4% DMSO or 20 μM aripiprazole for 12 hours at 37°C and 5% CO_2_. For protein extraction, 2 x 10^6^ cells were washed once in PBS and lysed in 100 μL of 1x Laemmli sample buffer with the addition of 1:20 β-mercaptoethanol (BIO-RAD). The lysates were boiled at 95°C for 5 minutes. Lysates were pelleted at 13,000 x g for 5 minutes, and 50 μL of lysate was run per SDS-PAGE gel. After transfer to nitrocellulose, the membrane was blocked with 5% bovine serum albumin (BSA) and incubated with a 1:2000 dilution of rabbit anti-Pan Actin IgG (Cell Signaling Technologies) and a 1:250 dilution of rabbit anti-phosphorylated Src family proteins (Tyr416; Cell Signaling Technologies). The nitrocellulose membrane was subsequently incubated with goat anti-rabbit IgG IRDye 680RD secondary antibodies (LI-COR Biosciences) at a 1:15,000 dilution. Western blots were imaged and analyzed using the Li-Cor Odyssey Fc imaging system and Image Studio (LI-COR Biosciences). The ratio of the fluorescence intensity measured in phosphorylated-Src relative to the Pan-Actin loading control was quantified from at least three biological replicates and graphed.

### Statistical analysis

Prior to conducting any statistical comparisons, data from each experiment was checked for normality using the Shapiro-Wilk normality test, and homogeneity of variances was tested with Bartlett’s test, with a P-value greater than 0.05 used to confirm the normal distribution of data. Statistical comparisons were performed by one-way analysis of variance (ANOVA) with Tukey post-tests or unpaired t-tests from at least three independent replicates. P values <  0.05 were considered significant.

## Results

### Aripiprazole alone, and not clozapine, olanzapine, or quetiapine, caused a loss of cell viability through apoptosis in differentiated HL-60 cells

**Fig 1:**
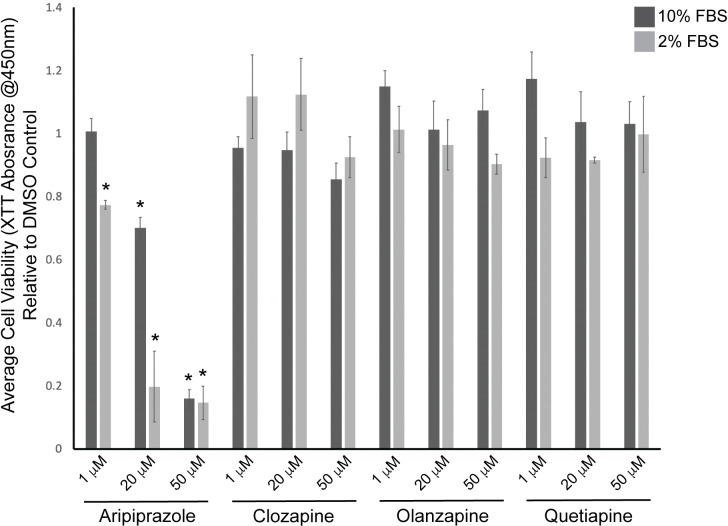
Aripiprazole, but not clozapine, olanzapine, or quetiapine cause loss of dHL-60 cell viability. dHL-60 cells were treated with 1 μM, 20 μM, or 50 μM in media containing 10% or 2% serum for 48 hours. Cell viability was measured by absorbance of XTT at 450 nm. The average absorbances relative to DMSO control were graphed from at least three replicates. Error bars indicate the standard error of the mean. *  indicates statistical significance via ANOVA with Tukey’s post-hoc test.

To determine if the atypical antipsychotics – aripiprazole, clozapine, olanzapine, and quetiapine – affect neutrophil cell viability, we tested the drugs on the differentiated neutrophil model cell line dHL-60 at concentrations of 1 μM, 20 μM, and 50 μM for 48 hours. Aripiprazole exhibited a dose-dependent loss of cell viability relative to DMSO controls. However, we did not observe any significant effects from clozapine, olanzapine, or quetiapine (Fig 1). Since serum proteins have been shown to decrease the bioavailability of compounds in the media, we treated the cells with atypical antipsychotic drugs in media containing 10% serum and 2% serum. Aripiprazole significantly reduced cell viability in 10% serum-containing media at the 20 μM and 50 μM concentrations but not at the 1 μM concentration. However, cells treated with aripiprazole in 2% serum resulted in decreased cell viability at all concentrations tested, with the effects enhanced due to the reduced amount of serum (Fig 1). However, we failed to observe any significant effects from clozapine, olanzapine, and quetiapine at either 10% or 2% serum conditions.

**Fig 2:**
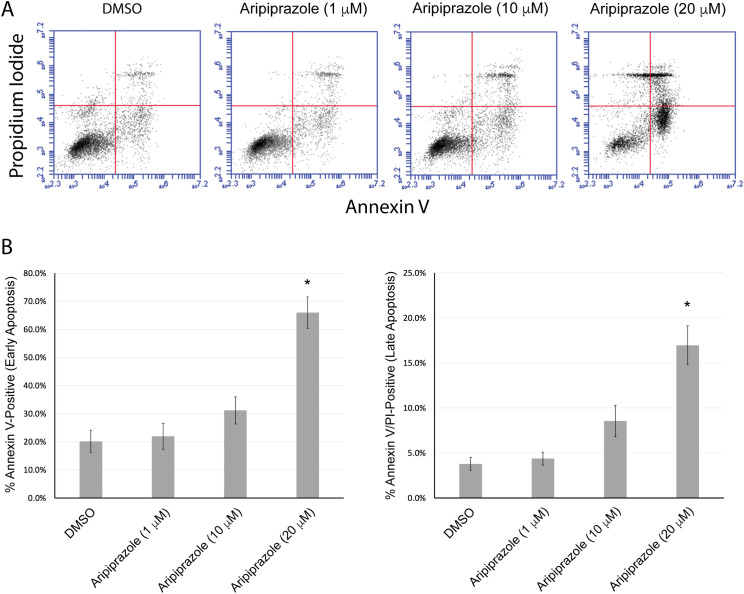
Aripiprazole exhibits a dose-dependent increase in dHL-60 cell apoptosis. Flow cytometry analysis of annexin V and propidium iodide staining in dHL-60 cells treated with 1 μM, 10 μM, or 20 μM aripiprazole for 24 hours in media containing 10% serum. A) Scatterplot of viable cells (lower left quadrant), early apoptotic cells (lower right quadrant), late apoptotic cells (upper right quadrant), and necrotic cells (upper left quadrant) from one representative replicate. At acquisition, 10,000 total cells were measured for each replicate. B) Graph representing the average percentage of early and late apoptotic cells from three independent biological replicates. Error bars indicate the standard error of the mean. *  indicates statistical significance via ANOVA with Tukey’s post-hoc test.

**Fig 3:**
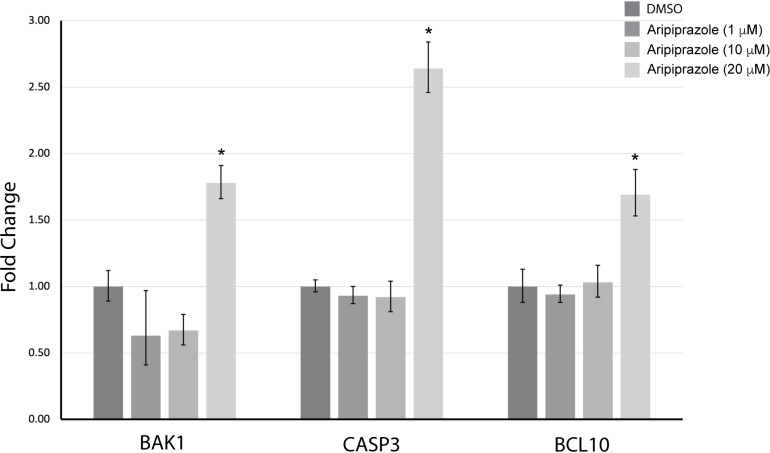
Aripiprazole induces an increase in pro-apoptotic genes in dHL-60 cells. RT-qPCR analysis of BAK1, caspase-3, and BCL10 mRNA expression in dHL-60 cells treated with 1 μM, 10 μM, or 20 μM aripiprazole for 24 hours. mRNA fold change was calculated using the 2^-ΔΔCq^ method. β-actin and RPL13a were used as reference genes. Data was quantified as described in Taylor et al. (2019). The mean Cq. values were normalized to the reference genes and graphed relative to DMSO controls. The data represent the mean + /- standard error of the mean from three independent replicates. *  indicates statistical significance via ANOVA with Tukey’s post-hoc test.

To further characterize aripiprazole’s effect on dHL-60 cells, we assessed apoptosis using the Annexin V/PI flow cytometry assay (Fig 2). Annexin V-positive cells indicate early apoptosis, while Annexin V and PI double positive cells indicate late apoptotic stages, characterized by the loss of plasma membrane integrity. Aripiprazole exhibited a dose-dependent increase in early and late apoptosis compared to DMSO controls in dHL-60 cells treated with 1 μM, 10 μM, and 20 μM concentrations after 24 hours (Fig 2A). While there was an apparent increase in the percentage of early and late apoptotic cells, only the 20 μM concentration was statistically significant (Fig 2B). To further characterize aripiprazole-induced apoptosis, we performed RT-qPCR to assess the expression of the pro-apoptotic genes Bcl2 antagonist/killer 1 (BAK1), caspase-3 (CASP3), and BCL10 in dHL-60 cells treated with 1 μM, 10 μM, and 20 μM aripiprazole for 24 hours (Fig 3). The expression of all three pro-apoptotic genes was significantly increased compared to the two internal reference genes, β-actin and ribosomal protein L13a. Caspase-3 exhibited the most significant increase compared to BAK1 and BCL10, and we did not observe any significant increase at concentrations below 20 μM aripiprazole. These data indicate that aripiprazole induces dHL-60 cell death through apoptotic mechanisms.

### Aripiprazole causes differentiated HL-60 cell death through dopamine receptor-independent mechanisms

**Fig 4:**
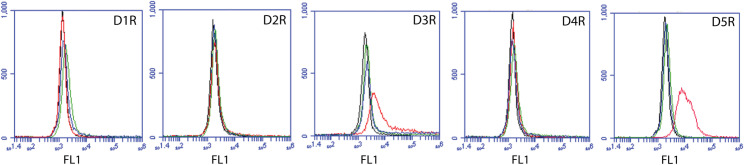
dHL-60 cells express dopamine receptors D3R and D5R, but not D1R, D2R, or D4R. Flow cytometry analysis of dopamine receptor surface expression on dHL-60 cells using polyclonal antibodies directed against extracellular epitopes of specific dopamine receptor subtypes. Representative histograms from three independent replicates illustrate the fluorescence intensity of unstained negative controls (black line), secondary antibody controls (blue line), IgG isotype controls (green line), and dopamine-specific receptors (red line).

Aripiprazole is unique in its ability to act as a partial agonist of dopamine receptors D2R, D3R, and D4R, while in some reports, aripiprazole can also act as an antagonist to the dopamine D2R receptor. But previous studies about dopamine receptor expression in primary neutrophils and dHL-60 cells remain unclear. Therefore, we characterized dopamine receptor surface expression using antibodies directed against the extracellular domains of each dopamine receptor subtype by flow cytometry (Fig 4). We identified the expression of dopamine receptors D3R and D5R in dHL-60 cells but did not detect the presence of receptors D1R, D2R, or D4R. These results indicate that dHL-60 cells express D1-like (D5R) and D2-like (D3R) dopamine receptor subtypes.

**Fig 5:**
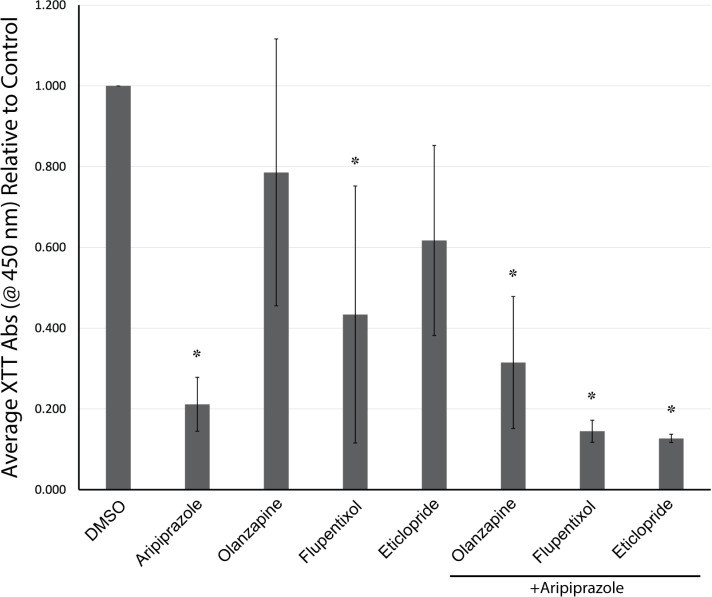
Dopamine receptor antagonists fail to inhibit aripiprazole-mediated loss of cell viability. dHL-60 cells were treated with 20 μM of the dopamine receptor antagonist olanzapine, flupentixol, or eticlopride in the absence or presence of aripiprazole for 48 hours in media containing 2% serum. The average absorbance relative to DMSO control was graphed from at least three replicates. Error bars indicate the standard error of the mean. *  indicates statistical significance via ANOVA with Tukey’s post-hoc test.

Confirmed dopamine receptor expression in dHL-60 cells suggests that aripiprazole’s apoptotic effects may be through dopamine receptor agonism or antagonism. To test this hypothesis, we performed cell viability assays in dHL-60 cells pre-treated with the dopamine receptor antagonist olanzapine, flupentixol, or eticlopride in the absence or presence of aripiprazole on cell viability (Fig 5). All treatments were at 20 μM since the concentration showed significant cytotoxicity in prior experiments. If aripiprazole causes loss of cell viability through dopamine receptor agonism, we predict that pre-treatment with dopamine receptor antagonists would block or mitigate aripiprazole-induced loss of cell viability. Interestingly, flupentixol, but not olanzapine and eticlopride alone, exhibited a significant loss of cell viability like aripiprazole. In addition, dHL-60 cells treated with aripiprazole in the presence of the antagonists did not alter the observed effects of aripiprazole on cell viability.

**Fig 6:**
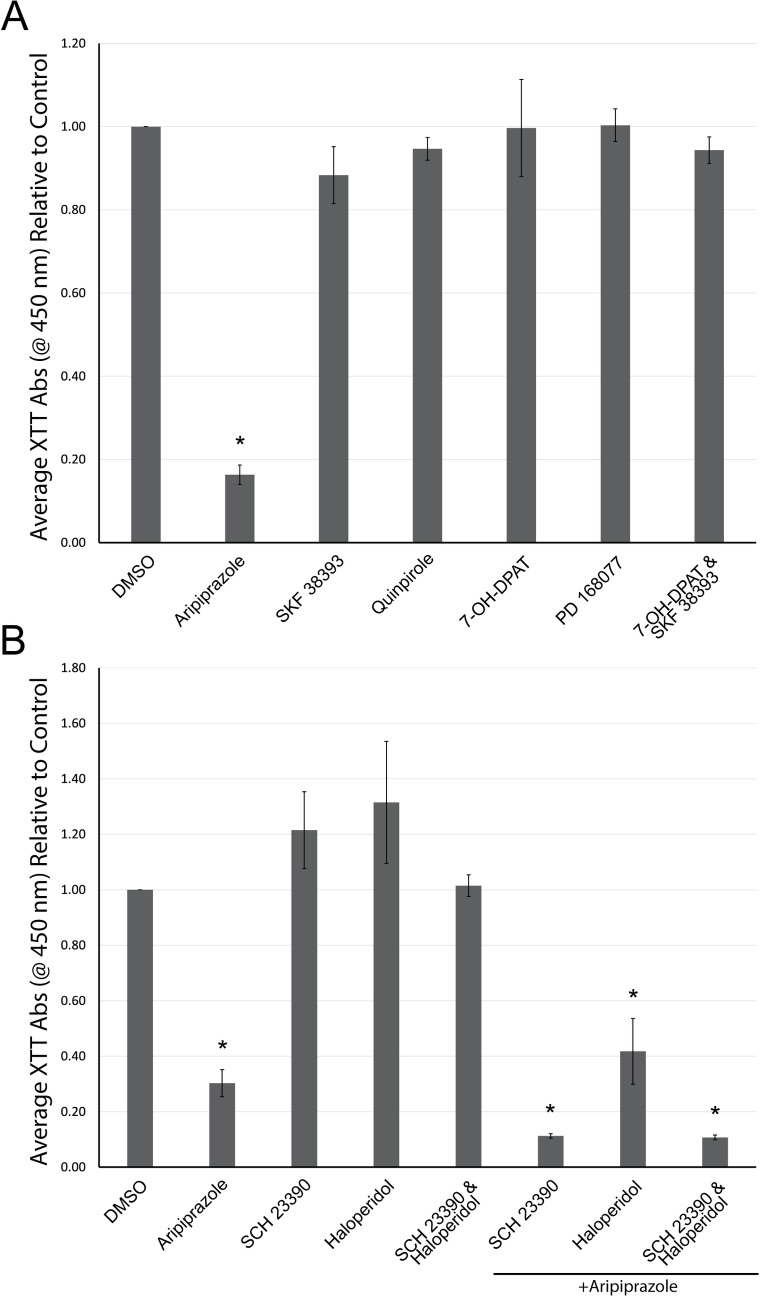
Dopamine receptor agonists and antagonists do not affect dHL-60 cells and aripiprazole-induced loss of cell viability. A) dHL-60 cells were treated with 20 μM of dopamine receptor agonists or B) antagonists in the absence or presence of aripiprazole for 48 hours in media containing 2% serum. The average absorbance relative to DMSO control was graphed from at least three replicates. Error bars indicate the standard error of the mean. *  indicates statistical significance via ANOVA with Tukey’s post-hoc test.

Dopamine receptor signaling is complex. D1-like receptors (D1R and D5R) activate Gαs, leading to the production of cAMP through adenylyl cyclase activation, whereas D2-like receptors (D2R, D3R, and D4R) inhibit adenylyl cyclase and cAMP signaling. Therefore, aripiprazole may be signaling through specific dopamine receptor subtypes since dHL-60 cells express D1-like and D2-like receptors. To test this hypothesis, we analyzed cell viability in dHL-60 cells treated with dopamine receptor subtype agonists and antagonists, as indicated in Table 1. dHL-60 cells treated with dopamine receptor-specific agonists displayed no significant decreases in cell viability, as seen with aripiprazole (Fig 6A). Moreover, dHL-60 cells treated with specific dopamine receptor antagonists in the presence or absence of aripiprazole did not rescue the loss of cell viability or cause loss of cell viability alone (Fig 6B). This data suggest that aripiprazole’s cytotoxicity is not through direct dopamine receptor agonism or antagonism but possibly through a dopamine receptor-independent mechanism.

**Fig 7:**
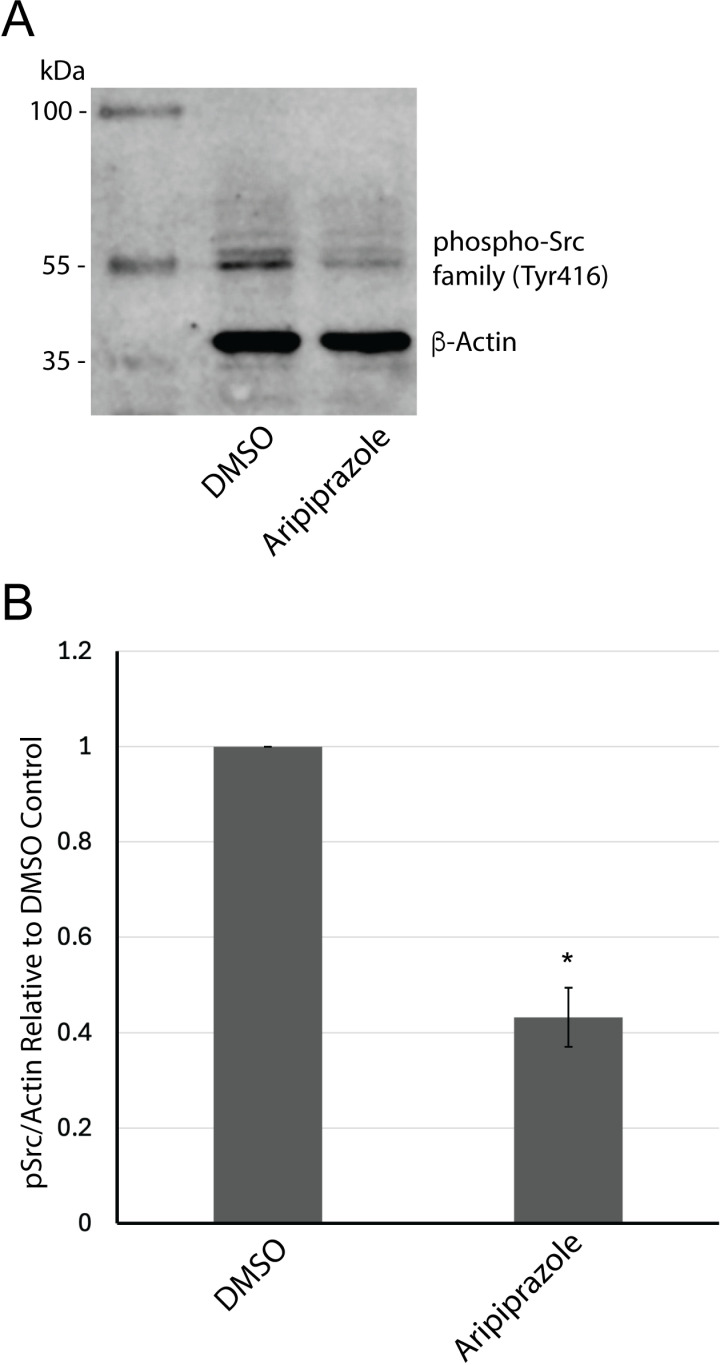
Aripiprazole significantly decreases phosphorylated Src (Y416) levels in dHL-60 cells. A) Western blot analysis of phosphorylated Src family proteins (Y416) and β-actin in dHL-60 cells treated with 20 μM aripiprazole for 12 hours in media containing 2% serum. B) Quantification of the average fluorescence intensity ratio of phosphorylated Src family proteins and β-actin relative to DMSO control from three biological replicates. *  indicates statistical significance via t-test.

To investigate a possible signaling mechanism, we characterized the effects of aripiprazole on phosphorylated Src family proteins, which are key kinases regulating cell survival, growth, and apoptosis. dHL-60 cells treated with 20 μM aripiprazole for 12 hours exhibited a significant reduction in the level of phosphorylated Src family proteins at tyrosine 416, within the activation loop of the kinase domain, relative to DMSO control-treated cells (Fig 7). This indicates that aripiprazole directly affects cell survival signaling proteins, which may be the cause of enhanced apoptosis in dHL-60 cells.

## Discussion

Our results show that aripiprazole, but not clozapine, olanzapine, or quetiapine, exhibits cytotoxicity in the neutrophil model HL-60 cell line through apoptotic mechanisms. Flow cytometry analysis of dopamine receptor expression confirmed the presence of dopamine receptors D3R and D5R. However, cell viability experiments using dopamine receptor-specific agonists and antagonists failed to replicate or rescue the aripiprazole-induced cytotoxicity, suggesting a dopamine receptor-independent mechanism. Further analysis demonstrated that aripiprazole treatment significantly reduced phosphorylated-Src kinase family protein levels, suggesting that aripiprazole exhibits cytotoxicity through the modulation of apoptotic signaling pathways.

Drug-induced neutropenia is a rare and severe side effect of some atypical antipsychotics that can be life-threatening. Proposed mechanisms for this condition include cytotoxicity by secondary metabolites [15] and genetic factors leading to adverse drug immune responses [44,45]. However, much remains unknown about the effects of these drugs on neutrophils, the first responders of innate immunity. Experiments with primary neutrophils are challenging due to their inability to be cultured for an extended period *in vitro*. Therefore, in our experiments, we investigated antipsychotic drug-induced neutropenia in the human promyelocytic cell line HL-60, which can be differentiated into neutrophil-like cells (dHL-60) and is commonly used as a human neutrophil model cell line [46,47].

In this study, we characterized the effects of four atypical antipsychotics on dHL-60 cell viability using the XTT assay at concentrations of 1 μM, 20 μM, and 50 μM for 48 hours. We report that dHL-60 cells treated with aripiprazole but not clozapine, olanzapine, or quetiapine exhibited a significant dose-dependent decrease in cell viability. We chose treatment concentrations of atypical antipsychotics in this range based on prior studies. For example, previous reports demonstrated the cytotoxic effects of clozapine on HL-60 cells using a range of 1 μM to 100 μM [17,48]. The concentrations of aripiprazole used in these experiments are higher than normally found in blood, which can be between 0.9 μM and 1.1 μM [49]. However, Aravagiri et al. (1999) report that olanzapine can accumulate at 10- to 30-fold higher concentrations in brain tissue, suggesting the same may be true for aripiprazole and other antipsychotic medications [50]. Initial experiments were performed in media containing 10% serum; however, many drugs readily bind to serum molecules and reduce the free drug concentration that can reach the receptor [51]. Therefore, we repeated the experiment in 2% serum to determine if there would be increases in the effectiveness of the treatments. Aripiprazole exhibited a significant decrease in cell viability similar to the 10% serum conditions, although no statistically significant effects were observed with clozapine, olanzapine, or quetiapine. Our results show a statistically significant difference in average XTT absorbance relative to control cells, and the lower serum concentration significantly enhances the loss in cell viability, with concentrations as low as 1 μM being statistically significant. Surprisingly, other antipsychotic medications did not affect the cells despite reports on their ability to induce neutropenia [25,26]. One explanation could be that clozapine, olanzapine, and quetiapine exert their effects through indirect mechanisms. For example, cytotoxic metabolites derived from clozapine may be responsible for neutrophil depletion [17,42,52,53]. These metabolites form by oxidation from activated neutrophils. Although activation markers were not analyzed in our assays, dHL-60 cells are inactive under the conditions presented in this research, and no activation agents, such as formyl-Met-Leu-Phe (fMLF), were added. We repeated previously published experiments to demonstrate that oxidized clozapine by hydrogen peroxide (H_2_O_2_) exhibits a significant increase in dHL-60 apoptosis, supporting the hypothesis that the metabolites of clozapine, and not clozapine itself, cause loss of cell viability in neutrophils and dHL-60s (S1 Fig).

Because loss of cell viability can occur by several distinct mechanisms, we demonstrate through flow cytometry analysis that aripiprazole induces cell apoptosis in a dose-dependent manner, with 20 μM causing a statistically significant increase in Annexin V- and PI-positive populations compared to control cells. Although the lower concentrations of 1 μM and 10 μM did not reach statistical significance, there was an observable increase in the percentage of apoptotic populations. To further substantiate these findings, RT-qPCR analysis demonstrated that 20 μM aripiprazole significantly increased the expression of pro-apoptotic genes BAK1, CASP3, and BCL10. These results align with a previous study demonstrating aripiprazole’s effects on the breast cancer cell line MCF-7 at similar concentrations [33]. The cells were incubated with aripiprazole for 24 hours in media containing 10% serum because trials performed in 2% serum or for longer incubation periods led to a complete loss of cell viability and integrity at the highest aripiprazole concentration, preventing reliable flow cytometry and RT-qPCR analysis. Therefore, we believe these conditions likely reduced free-drug concentration, possibly explaining why the lower concentrations did not show significance.

We hypothesized that aripiprazole induced apoptosis in dHL-60 cells through dopamine receptor-mediated mechanisms because, like most atypical antipsychotics, aripiprazole exerts its effects by agonizing and antagonizing dopamine receptors. Moreover, dopamine caused a dose and time-dependent induction of apoptosis in purified polymorphonuclear neutrophils (PMNs) from healthy patients and those with systemic inflammatory response syndrome [54]. However, the literature on dopamine receptor expression in neutrophils and dHL-60 cells is unclear. For instance, surface expression of D2R-D5R receptors has been reported in human primary neutrophils, with D3R and D5R expressed in all individuals and D2R and D4R expressed in a subset of individuals [8]. Immunohistochemistry studies have also reported D1R expression on isolated PMNs from healthy control patients [54]. Using RT-qPCR, D1R, D2R, D3R, and D5R expression was reported in ATRA-differentiated HL-60 cells [9], while only low levels of D3R were detected in primary neutrophils [55]. Our study used antibodies against the extracellular domains of dopamine-receptor subtypes to characterize surface expression in DMSO-differentiated HL-60 cells. Our results show that dHL-60 cells express D3R (a D2-like receptor subtype) and D5R (a D1-like receptor subtype) but not D1R, D2R, or D4R. These findings align with previous reports identifying D3R and D5R as the most highly and ubiquitously expressed dopamine receptors among isolated PMNs [8]. However, our results do not support the findings from molecular analyses. Dopamine receptor expression may vary based on individual donors or differentiation methods. For example, one previous study used ATRA instead of DMSO as the differentiation agent [55], potentially resulting in different biological properties of the HL-60 cell line, which has been reported for other neutrophil mechanisms [56]. Moreover, it’s possible that select antibodies were non-specific or ineffective under our experimental conditions. A systematic study employing flow cytometry and RT-qPCR on purified primary neutrophils and differentiated HL-60 cells is necessary to reconcile these conflicting reports. Nevertheless, our data suggest that DMSO-differentiated HL-60 cells express D1-like and D2-like dopamine receptor subtypes, supporting the hypothesis that aripiprazole may be acting through these receptors.

To test whether aripiprazole causes loss of cell viability through dopamine receptor agonism, we attempted to incubate dHL-60 cells with aripiprazole that were pre-treated with dopamine receptor antagonists olanzapine, flupentixol or eticlopride. Yet, these combinations led to a further loss of cell viability. Interestingly, flupentixol alone caused a statistically significant loss of cell viability, akin to aripiprazole. Next, we treated dHL-60 cells with various specific dopamine receptor subtype agonists and antagonists. We could not replicate the aripiprazole-induced loss of cell viability or rescue it with any of the compounds tested to support the conclusion that aripiprazole is signaling through a specific dopamine receptor subtype. Aripiprazole may exhibit complex signaling mechanisms, resulting in the heterodimerization of dopamine receptors, as has been reported [7]. Consequently, these data suggest that aripiprazole exhibits dHL-60 cytotoxicity through a dopamine receptor-independent mechanism. The effects of aripiprazole, and possibly the combinatorial effects from the co-incubation of olanzapine, flupentixol, or eticlopride, could be through other receptor-signaling mechanisms. For example, aripiprazole, olanzapine, and flupentixol can also signal through serotonin 5-HT_2A_ receptors [11], and thus, no effect would be observed by trying to block or mimic dopamine receptor signaling.

Surprisingly, only flupentixol could mimic aripiprazole-induced cytotoxicity. Flupentixol is a dopamine antagonist, while aripiprazole is a partial dopamine agonist and antagonist. One possible explanation stems from a recent report characterizing flupentixol’s anti-cancer properties through PI3K inhibition [57]. The PI3K/AKT pathway regulates cell proliferation, differentiation, adhesion, and survival [58]. Upregulation of this pathway is commonly associated with tumorigenesis in various cancers, including leukemia, breast, and non-small cell lung cancers (NSCLCs). Moreover, aripiprazole can inhibit Src activity in vitro, reduce viability in several cancer cell lines, and induce apoptosis in U251 glioma cells [32]. Because dHL-60 cells originate from a myeloid leukemia cell line, it’s plausible that aripiprazole exhibits anti-cancer mechanisms like flupentixol, especially at higher concentrations. To test this hypothesis, we showed a significant reduction in the levels of phosphorylated Src family proteins at tyrosine 416 in dHL-60 cells, which supports previous literature that aripiprazole may bind directly to Src kinase *in vitro* and regulate cell survival and apoptosis [32]. Our findings support the hypothesis that aripiprazole may induce neutropenia through receptor-independent cytotoxicity. However, understanding the exact mechanisms requires further study.

Aripiprazole is an enigmatic drug. Our results support previous clinical studies of aripiprazole causing neutropenia and leukocytopenia [28–30]. Moreover, in multiple cancer cell lines, aripiprazole inhibited cell proliferation and induced apoptosis through upregulation of pro-apoptotic genes and inhibition of pro-tumorigenic signaling pathways, such as phosphorylated Src, phosphorylated phosphatidylinositol 3-kinase (PI3K), and phosphorylated signal transducer and activator of transcription 3 (STAT3) [32,33]. However, in rodent models and neuronal cell lines, aripiprazole exhibits neuroprotective effects. For example, several reports demonstrate that aripiprazole increases brain-derived neurotrophic factor (BDNF) and decreases pro-apoptotic processes in neuroblastoma cells, rat neuron cultures, and activated microglia [59–61]. Aripiprazole reduces overall oxidative stress in several rat models for depression, mania, and autism [62–64]. Finally, aripiprazole has anti-inflammatory properties where it’s been shown to reduce many pro-inflammatory genes and cytokines in randomized clinical trials, neuronal cell lines, and LPS-activated microglia [65–67]. Several of the studies indicate receptor-independent effects of aripiprazole. To date, no clear mechanism explains the apparent dichotomy of aripiprazole’s functions. One possible explanation is that aripiprazole has been suggested to be functionally selective at multiple receptors [68,69], meaning the drug can interact with a single receptor to elicit multiple mechanisms of action and that this change in mechanism may be driven by the cellular environment surrounding the ligand and the specific receptor. For example, in D2_L_-transfected MES23.5 cells, a neuroblastoma cell line, aripiprazole functions as an antagonist yet exhibits partial agonism towards D2_L_-transfected Chinese hamster ovary (CHO) and human embryonic kidney (HEK-293) cells [69].

Although aripiprazole has been associated with neutropenia in clinical case studies and cytotoxicity in tumor cell lines, there has yet to be an in vitro characterization of its effects on primary human neutrophils. HL-60 cells are a promyelocytic leukemia line characterized predominantly by c-Myc gene amplification [70]. The differentiation of HL-60 cells into granulocytes by DMSO (dHL-60) is driven by the downregulation of c-Myc gene expression and the upregulation of several kinases involved in intracellular signaling [71,72]. Upon differentiation, HL-60 cells express receptors necessary for cell polarization, chemotaxis, reactive oxygen species (ROS) production, NETosis, and phagocytosis, mimicking primary human neutrophils. However, the expression levels and functional responses of these receptors are generally lower than those observed in primary human neutrophils [5]. Therefore, while differentiated HL-60 cells provide a valuable in vitro model system for studying neutrophil processes, they do not fully replicate the complexity, maturity, and functionality of primary human neutrophils. Therefore, whether aripiprazole exhibits similar effects to primary human neutrophils in vitro remains to be investigated. Nevertheless, our results suggest the potential for aripiprazole-induced cytotoxicity. Thus, routine blood monitoring is recommended, especially in patients who have a history of neutropenia with other antipsychotics.

This study reveals that aripiprazole exhibits cytotoxicity through an apoptotic mechanism in differentiated HL-60 cells. Flow cytometric assessments of D3R and D5R dopamine receptor expression and cell viability analyses after treatment with dopamine receptor agonists and antagonists in dHL-60s revealed that aripiprazole induces cell death through mechanisms independent of dopamine receptor signaling, likely through regulation of Src family kinase pathways. These results will strengthen the current understanding of the varied side effectsof atypical antipsychotics and will help in understanding the probable mechanisms that contribute to common non-specific drug effects of this class of medications.

## Supporting Information

S1 Fig Oxidized clozapine exhibits loss of cell viability in dHL-60 cells. Flow cytometry analysis of Annexin V and Propidium Iodide staining in dHL-60 cells treated with DMSO, 20 μM clozapine, 10 μM H_2_O_2_, or 20 μM clozapine and 10 μM H_2_O_2_ combined. A) Scatterplot of viable cells (lower left quadrant), early apoptotic cells (lower right quadrant), late apoptotic cells (upper right quadrant), and necrotic cells (upper left quadrant) from one representative replicate. At acquisition, 10,000 total cells were measured for each replicate. B) Graph representing the average percentage of early and late apoptotic cells from three independent biological replicates. Error bars indicate the standard error of the mean. *  indicates statistical significance via ANOVA with Tukey’s post-hoc test.
(TIF)

S1 Appendix Raw data from all biological replicates for Figs 1–7. (XLSX)
