# Supplementary figures and images for: Characterization of the atypical antipsychotic drug aripiprazole cytotoxicity in the neutrophil model cell line HL-60
Source: PLoS One. 2025 Feb 12;20(2):e0318878. doi: 10.1371/journal.pone.0318878 (PMC11819542; doi:10.1371/journal.pone.0318878)

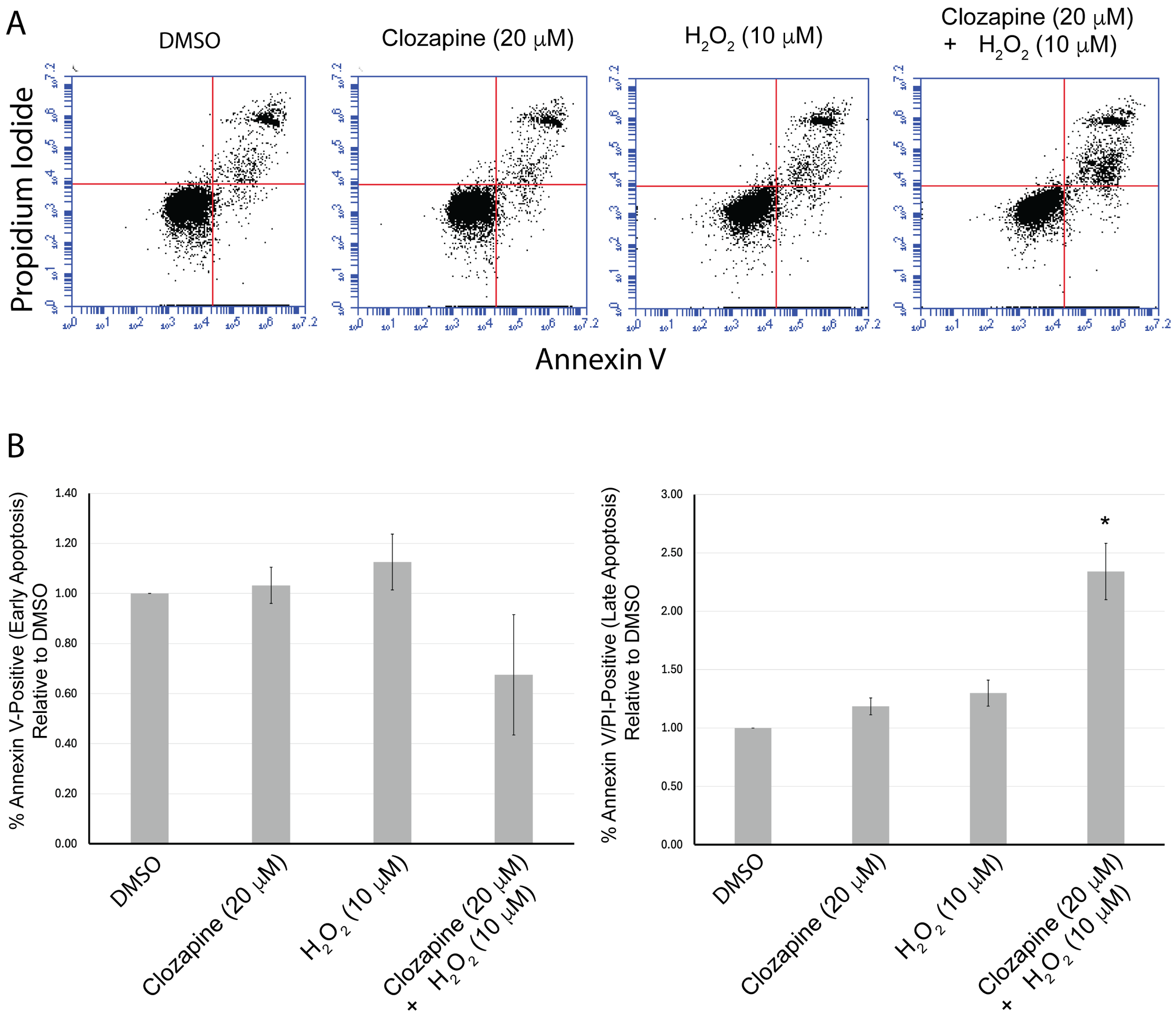

Supplement: S1 Fig — Flow cytometry analysis of Annexin V and Propidium Iodide staining in dHL-60 cells treated with DMSO, 20 μM clozapine, 10 μM H2O2, or 20 μM clozapine and 10 μM H2O2 combined. A) Scatterplot of viable cells (lower left quadrant), early apoptotic cells (lower right quadrant), late apoptotic cells (upper right quadrant), and necrotic cells (upper left quadrant) from one representative replicate. At acquisition, 10,000 total cells were measured for each replicate. B) Graph representing the average percentage of early and late apoptotic cells from three independent biological replicates. Error bars indicate the standard error of the mean. * indicates statistical significance via ANOVA with Tukey’s post-hoc test. (TIF) [file pone.0318878.s001.tif]
